# Worldwide trends in quantity and quality of published articles in the field of infectious diseases

**DOI:** 10.1186/1471-2334-5-16

**Published:** 2005-03-21

**Authors:** Ioannis A Bliziotis, Konstantinos Paraschakis, Paschalis I Vergidis, Antonia I Karavasiou, Matthew E Falagas

**Affiliations:** 1 Alfa Institute of Biomedical Sciences(AIBS), Athens, Greece; 2 Alfa HealthCare, Athens, Greece; 3 Department of Medicine, Tufts University School of Medicine, Boston, Massachusetts, USA

## Abstract

**Background:**

Trying to confront with the widespread burden of infectious diseases, the society worldwide invests considerably on research. We evaluated the contribution of different world regions in research production in Infectious Diseases.

**Methods:**

Using the online Pubmed database we retrieved articles from 38 journals included in the "Infectious Diseases" category of the "Journal Citation Reports" database of the Institute for Scientific Information for the period 1995–2002. The world was divided into 9 regions based on geographic, economic and scientific criteria. Using an elaborate retrieval system we obtained data on published articles from different world regions. In our evaluation we introduced an estimate of both quantity and quality of research produced from each world region per year using: (1) the total number of publications, (2) the mean impact factor of publications, and (3) the product of the above two parameters.

**Results:**

Data on the country of origin of the research was available for 45,232 out of 45,922 retrieved articles (98.5 %). USA and Western Europe are by far the most productive regions concerning publications of research articles. However, the rate of increase in the production of articles was higher in Eastern Europe, Africa, Latin America and the Caribbean, and Asia during the study period. The mean impact factor is highest for articles originating in the USA (3.42), while it was 2.82 for Western Europe and 2.73 for the rest of the world (7 regions combined).

**Conclusion:**

USA and Western Europe make up a striking 80% of the world's research production in Infectious Diseases in terms of both quantity and quality. However, all world regions achieved a gradual increase in the production of Infectious Diseases articles, with the regions ranking lower at present displaying the highest rate of increase.

## Background

Infectious diseases constitute a major health problem both in developed and developing countries. Old and emerging infectious diseases contribute substantially to morbidity and mortality worldwide. For this reason, the society invests considerably on infectious diseases research, in order to achieve scientific progress and develop new therapeutic interventions.

The research productivity by various world regions has been studied for several biomedical fields. In general, the USA and Western Europe are the leaders of global biomedical research, although their relative contribution varies for different fields of research [1-4]. Several studies have focused on the scientific production of European Union's countries, in various biomedical fields, including Infectious Diseases [5-7]. However, the literature lacks studies estimating the quantity and quality of worldwide research production in Infectious Diseases. The purpose of our study was to evaluate the contribution of different world regions in scientific research in the field of Infectious Diseases. We also evaluated the trends in quality and quantity of published articles from different world regions.

## Methods

We used the electronic PubMed database [8] and data from the Journal Citation Reports (JCR) database of the Institute for Scientific Information (ISI) [9]. We searched for articles included both in the "Infectious Diseases" category of the JCR and in PubMed database. Articles published prior to 1995 were not included in the analysis, because the full address of the authors of the papers was frequently not registered in PubMed prior to this year. Because JCR had available data up to the year 2002 at the time of our analysis, our data collection and evaluation refers to the period 1995–2002. A total of 38 journals were included. Two independent investigators conducted the data collection (IAB, PIV).

For the purpose of this study, the world was divided into 9 regions based on a combination of geographic, economic and scientific criteria [10]. The 9 regions are Western Europe, Eastern Europe, United States of America (USA), Canada, Latin America and the Caribbean, Africa, Japan, Asia (excluding Japan), and Oceania.

In our search of different fields in the Pubmed database we used a phrase consisting of four parts joined together by the so-called Boolean operators, i.e. AND, OR, and NOT. Each search was limited to a specific year using the "Limits" function, which is incorporated in the search engine. We only analyzed data on original articles and reviews, excluding publication types, such as letters, editorials, and news reports. For example in order to search for articles published in the "Journal of Infectious Diseases" and whose first author's address was in Europe, we used the following text: *Journal of Infectious Diseases [journal] AND journal article [pt] AND (Andorra [AD] OR Austria [AD] OR... Wales [AD]) NOT (Australia [AD] OR Canada [AD] OR...)*. In the first parenthesis of the search phrase, the countries of the implicated region are included. In the second parenthesis, after the word NOT, certain addresses are excluded in order to avoid double counting.

Subsequently, the results of these searches (the number of articles produced by each world region in a specific journal within a year) were summed up. For confirmation, the sum of articles produced by all different world regions in a journal, was compared to the actual total number of articles published in that journal for a specific year. This number was obtained from PubMed without using any address limits. Using this methodology we were able to cross-examine missed or unretrieved addresses. This occurred occasionally, in cases of articles with no address registered, and in cases of articles where only the affiliated institution or the city (not the country) was recorded. If less than 5% of the total articles of a specific journal during a year had missing addresses, we did not include these articles in our calculations, assuming that the numerical error was not significant. On the other hand, if more than 5% of the total articles of a specific journal during a year, had missing addresses, we performed searches for the author's address by checking other articles of the same author within the same year.

The number of published articles was considered as an index of quantity of research productivity. The mean impact factor of the published articles was considered as an index of quality of research productivity. Finally, the product of the number of articles published in a journal multiplied by the impact factor of the journal, for the year studied, was considered as an index evaluating combined the quantity and quality of research productivity. The sum of these products from all journals, for each world region within a year, was named "total product" for each region within the studied year. The impact factor for each journal was obtained from the JCR database of the ISI.

To further evaluate factors associated with the research published in Infectious Diseases journals we used relevant "World Development Indicators" [11] from the online databases of the World Bank. The research productivity of different world regions (estimated by the "total product") was evaluated in relation to total population, gross domestic product (GDP) in standard 1995 US dollars, and gross national income (GNI) per capita (Atlas method).

We used the absolute figures and the average annual rates of increase of scientific output (research productivity) of different world regions to calculate future performance using a projection model. Also, we performed correlation statistical analysis of the absolute numbers of published articles between the different world regions during the years of the study period (1995–2002) using Pearson correlation testing. In addition, we performed correlation statistical analysis to examine the research productivity of the specified world regions compared with the total world production.

## Results

The journals that were included in our analysis are shown in Table 1. Using the methodology described above, we managed to retrieve and categorize 45,232 out of 45,922 articles, (98.5%) from the implicated journals indexed in Pubmed during the study period. The total production of articles in each defined world region, as well as the relative contribution of each region to the total production in the field of Infectious Diseases, is displayed in Table 2. As shown in this table, USA and Western Europe are by far the most productive regions (79.8% of the articles published worldwide, throughout the whole period studied, came from these two regions). As expected, the difference between the USA and Western Europe increases when both the number of articles and impact factor are taken into account, due to the higher impact factor that USA had throughout the study period. In the years 2000–2002 Western Europe's production exceeded that of the USA, although USA researchers produced more articles in all previous years. The last column shows the "total product" of research published in the field of Infectious Diseases for each region for the whole study period.

**Table 1:** Title of journals included in the field of Infectious Diseases of the Institute for Scientific Information (ISI) indexed both by ISI and PubMed.

**Title of journal**	**Study period**
AIDS	1995 – 2002
AIDS Patient Care STDS	2002
AIDS Research and Human Retroviruses	1995 – 2002
American Journal of Infection Control	1995 – 2002
Antiviral Therapy	2000 – 2002
BMC Infectious Diseases	2002
Clinical and Diagnostic Laboratory Immunology	1995 – 2002
Clinical Infectious Diseases	1995 – 2002
Clinical Microbiology and Infection	2002
Current Opinion in Infectious Diseases	2000 – 2002
Diagnostic Microbiology and Infectious Disease	1995 – 2002
Emerging Infectious Diseases	1995 – 2002
Epidemiology and Infection	1995 – 2002
European Journal of Clinical Microbiology and Infectious Diseases	1995 – 2002
Infection	1995 – 2002
Infection and Immunity	1995 – 2002
Infection Control and Hospital Epidemiology	1995 – 2002
Infectious Agents and Disease	1995 – 1996
Infectious Disease Clinics of North America	1995 – 2002
International Journal of Antimicrobial Agents	2000 – 2002
International Journal of Hygiene and Environmental Health (prior to 1999: Zentralblatt fur hygiene und umweltmedizin)	1997 – 2002
International Journal of STD & AIDS	1995 – 2002
International Journal of Tuberculosis and Lung Disease	1998 – 2002
JAIDS-Journal of Acquired Immune Deficiency Syndromes (prior to 1998: Journal of Acquired Immune Deficiency Syndromes and Human Retrovirology)	1995 – 2002
Japanese Journal of Infectious Diseases	2000 – 2002
Journal of Antimicrobial Chemotherapy	1995 – 2002
Journal of Hospital Infection	1995 – 2002
Journal of Human Virology	2001 – 2002
Journal of Infection	1995 – 2002
Journal of Viral Hepatitis	1997 – 2002
Leprosy Review	1997 – 2002
Microbial Drug Resistance	1997 – 2002
Pediatric AIDS and HIV Infection	1997
Pediatric Infectious Disease Journal	1995 – 2002
Scandinavian Journal of Infectious Diseases	1995 – 2002
Sexually Transmitted Diseases	1995 – 2002
Sexually Transmitted Infections (prior to 1997: Genitourinary Medicine)	1999 – 2002
The Journal of Infectious Diseases	1995 – 2002

**Table 2:** Number of articles published in journals included in the "Infectious Diseases" category of "Journal Citation Report" database and indexed by PubMed, from different world regions, for the period 1995–2002.

	Number of articles **(% percentage within a calendar year)**									
**WORLD AREAS**	**1995**	**1996**	**1997**	**1998**	**1999**	**2000**	**2001**	**2002**	***1995–2002***	1995–2002*

**USA**	2118 **(46.98)**	2078 **(45.09)**	2162 **(43.66)**	2425 **(42.86)**	2346 **(40.27)**	2530 **(38.92)**	2543 **(38.48)**	2481 **(37.76)**	*18683 ****(41.30)***	*63804*
**Western Europe**	1673 **(37.11)**	1804 **(39.14)**	1863 **(37.62)**	2136 **(37.75)**	2248 **(38.59)**	2577 **(39.65)**	2579 **(39.03)**	2539 **(38.64)**	*17419 ****(38.51)***	*49033*
**Asia (excluding Japan)**	175 **(3.88)**	160 **(3.47)**	235 **(4.75)**	317 **(5.60)**	316 **(5.42)**	376 **(5.78)**	413 **(6.25)**	437 **(6.65)**	*2429 ****(5.37)***	*5927*
**Japan**	106 **(2.35)**	141 **(3.06)**	177 **(3.57)**	135 **(2.39)**	226 **(3.88)**	258 **(3.97)**	273 **(4.13)**	260 **(3.96)**	*1576 ****(3.48)***	*4113*
**Canada**	155 **(3.44)**	126 **(2.73)**	151 **(3.05)**	179 **(3.16)**	181 **(3.11)**	194 **(2.98)**	183 **(2.77)**	207 **(3.15)**	*1376 ****(3.04)***	*4510*
**Latin America and Caribbean**	76 **(1.69)**	82 **(1.78)**	101 **(2.04)**	127 **(2.24)**	130 **(2.23)**	154 **(2.37)**	173 **(2.62)**	189 **(2.88)**	*1032 ****(2.28)***	*2978*
**Oceania**	89 **(1.97)**	108 **(2.34)**	111 **(2.24)**	138 **(2.44)**	142 **(2.44)**	155 **(3.97)**	144 **(2.18)**	145 **(2.21)**	*1032 ****(2.28)***	*3153*
**Africa**	70 **(1.55)**	66 **(1.43)**	97 **(1.96)**	137 **(2.42)**	159 **(2.73)**	151 **(2.32)**	177 **(2.68)**	153 **(2.33)**	*1010 ****(2.23)***	*2913*
**Eastern Europe**	46 **(1.02)**	44 **(0.95)**	55 **(1.11)**	64 **(1.13)**	78 **(1.34)**	105 **(1.62)**	123 **(1.86)**	160 **(2.43)**	*675 ****(1.49)***	*1325*
***Total***	*4508 ****(100)***	*4609 ****(100)***	*4952 ****(100)***	*5658 ****(100)***	*5826 ****(100)***	*6500 ****(100)***	*6608 ****(100)***	*6571 ****(100)***	*45232 ****(100)***	*137756*

*Number of articles published multiplied by their impact factor

We observed a continuous increase in the production of research articles from all world regions during the period 1995–2002 (Table 2). There was a strong and statistically significant correlation between the absolute numbers of published articles between the different world regions during the years of the study period (1995–2002). The median (range) of the Pearson correlation test values between comparisons of 36 possible couples of the specified 9 world regions was 0.88 (0.48 – 0.99). Thirty of 36 comparisons had statistical significance at levels < 0.05 (24 of them had statistical significance at levels < 0.01). The comparisons that did not have statistical significance (p > 0.05) were between world regions with relative small numbers of published articles in the field of Infectious Diseases.

In addition, a strong and statistically significant correlation was noted between the annual research production of the specified world regions with that of the total world production; specifically the median (range) of Pearson correlation test results of these analyses were 0.94 (0.70–0.99). However, the rate of increase of research productivity in the field of Infectious Diseases was higher in Eastern Europe, Africa, Latin America and the Caribbean, and Asia. Using a projection model we estimated that these regions would reach USA's production level in 23 years and Western Europe's production level in 29 years, provided that each region maintains the average rate of increase of research production achieved in the 8-year-period studied.

Table 3 presents the mean impact factor of published articles in the field of Infectious Diseases for each region in the studied years. A mean value of the impact factor is also presented for the whole 8-year-period. The mean impact factor, for the whole period, is highest for articles originating in the USA. Interestingly, Canada ranks second and Western Europe ranks sixth regarding the mean impact factor of published articles. Eastern Europe has the lowest mean impact factor among all world regions.

**Table 3:** Mean impact factor of articles published in journals included in the "Infectious Diseases" category of "Journal Citation Report" database and indexed by Pubmed, from different world regions, for the period 1995–2002.

	**Mean impact factor**								
**WORLD AREAS**	**1995**	**1996**	**1997**	**1998**	**1999**	**2000**	**2001**	**2002**	***1995–2002 *(25^th^percentile, median, 75^th^percentile)**

**USA**	3.08	3.44	3.12	3.26	3.47	3.60	3.71	3.54	***3.42 (2.29 3.51 4.21)***
**Canada**	2.88	3.71	2.91	3.00	3.29	3.48	3.49	3.43	***3.28 (2.08 3.20 4.18)***
**Oceania**	2.69	3.17	3.01	2.84	2.75	3.53	3.28	2.99	***3.05 (1.79 2.80 4.18)***
**Africa**	2.83	2.87	3.12	2.46	3.00	2.87	3.01	2.90	***2.89 (1.63 2.20 4.18)***
**Latin America and Caribbean**	2.62	3.13	2.69	2.84	2.87	2.97	2.70	3.14	***2.89 (1.77 2.63 4.04)***
**Western Europe**	2.46	2.91	2.56	2.68	2.85	2.97	3.02	2.89	***2.82 (1.41 2.36 3.93)***
**Japan**	2.77	3.23	2.80	2.85	2.28	2.57	2.32	2.59	***2.61 (1.35 2.52 4.03)***
**Asia (excluding Japan)**	2.28	2.61	2.37	2.47	2.46	2.44	2.41	2.47	***2.44 (1.34 2.01 3.24)***
**Eastern Europe**	1.69	2.12	2.12	1.83	2.15	2.26	1.89	1.77	***1.96 (1.20 1.58 2.29)***
***Mean (for all regions)***	***2.77***	***3.17***	***2.83***	***2.93***	***3.06***	***3.18***	***3.21***	***3.10***	

**Figure 1:**
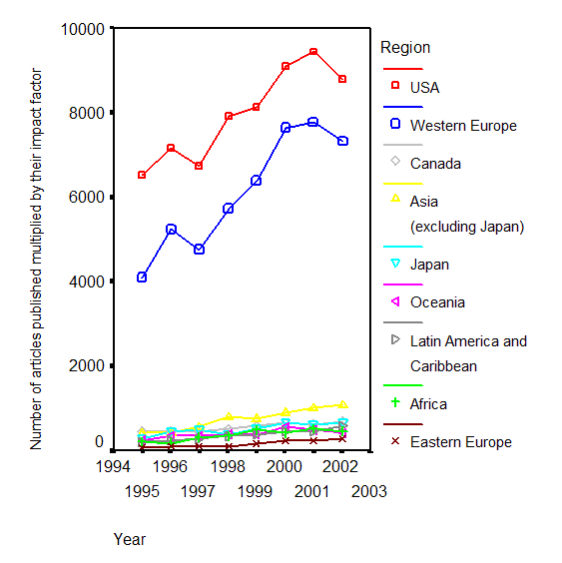
Graph displaying the worldwide trends of "total product" of research productivity (number of articles published multiplied by their impact factor) in Infectious Diseases, for different world regions, in the period 1995–2002.

Figure 1 depicts the worldwide trends of research productivity in the period 1995–2002. USA ranks first among all studied world regions, even during the period 2000–2002 in which investigators from Western Europe published a greater number of articles than investigators from USA. Eastern Europe had the most significant relative growth in the "total product" of research between 1995 and 2002.

Table 4 presents the quality and quantity of published research adjusted for the regional population and to the gross national income per capita (GNIPC). Specifically, it presents the ratio of scientific "total product" per population divided by the gross national income per capita for each region annually and the respective mean ratio for the whole period. USA and Canada are on the top of this list regarding the cumulative production of research in Infectious Diseases during the period 1995–2002. Interestingly, with the aforementioned adjustments Oceania ranks third on this list.

**Table 4:** Research output of different world areas, published in journals included in the category of "Infectious Diseases" of the Institute for Scientific Information (ISI), adjusted for population and gross national income per capita (GNIPC).

	**Number of publications multiplied by the impact factor per million of population divided by the GNIPC (in 10,000 1995 US dollars per capita)**								
**WORLD AREAS**	**1995**	**1996**	**1997**	**1998**	**1999**	**2000**	**2001**	**2002**	**Average**

**USA**	8.8	9.4	8.4	9.5	9.4	10.1	10.5	9.6	9.5
**Canada**	7.7	7.9	7.1	8.4	8.8	9.5	8.9	9.6	8.5
**Oceania**	5.3	7.4	6.9	7.8	7.4	10.2	8.5	7.6	7.6
**Africa**	4.2	3.8	6.0	6.4	8.8	7.7	9.1	7.4	6.7
**Western Europe**	4.5	5.7	5.0	5.9	6.4	7.4	7.4	6.9	6.2
**Asia (excluding Japan)**	1.4	1.3	1.7	2.3	2.2	2.5	2.6	2.7	2.1
**Latin America & the Caribbean**	1.2	1.5	1.5	1.9	2.0	2.4	2.4	3.1	2.0
**Eastern Europe**	0.8	0.9	1.2	1.2	1.6	2.1	2.0	2.4	1.5
**Japan**	0.6	0.8	0.9	0.7	0.9	1.2	1.1	1.2	0.9

## Discussion

Our study shows that USA and Western Europe make up a striking 80% of the world's research production in terms of both quantity and quality of articles published in Infectious Diseases journals. In addition, our study shows that scientific publications in Infectious Diseases journals increased from 1995 through 2002. The product of the number of published articles multiplied by the impact factor of the journals ("total product"), an index that estimates combined the quantity and quality of produced publications, also increased during the study period. The increased number of published articles in the Infectious Diseases journals, during the study period, is mainly attributed to the introduction of new titles of journals as well as an increase of the number of articles published in some of the journals in the field; both of these trends are mainly the result of increased demand for publishing due to increased production of research data [12,13].

Another interesting finding in our study was the relative reduction in research productivity of the USA compared to the rest of the world. This finding was also observed in the past by other investigators including the U.S. share of research articles in the leading basic and clinical research articles [14,15]. These observations may reflect mainly the improvement of the scientific output, including biomedical research, by several world areas as a result of the general improvement of their economic indices rather than absolute worsening of these factors in the USA.

We provide some data about the relative contribution in research productivity of different world regions in the field of Infectious Diseases. This quantitative data may be used in comparing the productivity of areas of the world with diverse economic status and priorities for funding of different social needs. In addition, our data may be useful as baseline information in evaluating the return of investment on research in Infectious Diseases in areas of the world where this is needed most, i.e. in the developing countries. Specifically, our data show that USA and Canada are the most productive regions when population and GNIPC are taken into account. However, it is interesting that Africa ranks fourth in research productivity when adjustments for these two factors are made. When interpreting this result, one should take into account that a large part of the research originating from this region is the result of multinational/multiregional collaborations, a fact that was not evaluated in this study. Nevertheless, our analysis shows that articles produced by investigators in Africa represent an important scientific contribution to the field of Infectious diseases, due to the very low GNIPC of the area, as well as a satisfactory return of the resources invested for Infectious Diseases research in the area.

Our study has several limitations in both the collection and interpretation of data. First, we used JCR criteria for including medical journals in the study. Articles published in non JCR-cited journals were not included, although they contribute to scientific production [16]. Moreover, we used the JCR impact factor. Although the impact factor has often been criticized as a tool for measuring scientific research quality [17-19], thus far it has not been replaced by any other worldwide-accepted method. JCR uses several criteria in order to include a journal in its databases, and up today the impact factor represents the best method of biomedical journal categorization [20-22]. Also, we used the PubMed, which is an easily accessible and widely used database. Nevertheless, some scientific articles are not included in this database and consequently were not analyzed in our study. In addition, in PubMed only the address of the first author is registered; thus studies that were created by multinational/multi-regional cooperation were counted as originating from only one region of the world. Another problem with the collection of data was associated with the fact that the search system we created was not able to retrieve the addresses of all articles. However, we managed to retrieve 98.5% of all published articles by performing meticulous searches for the address of the first author. Therefore, we assumed that the number of missed articles did not significantly affect our study results.

Another limitation is associated with the division of the world into different regions. Our categorization takes into account geographic, economic, and, most importantly, scientific criteria but despite that, alternative approaches would also be appropriate. For example, Canada could be grouped together with USA, and Japan could be studied together with the other Asian countries. Nevertheless, Canada and Japan represent powerful autonomous scientific world regions and thus we examined them as separate regions. In addition, when interpreting the results, one should take into account that many articles regarding infectious diseases are published in journals of other JCR categories such as "Medicine, General and Internal", "Medicine, Research and Experimental", "Virology", and "Parasitology" and not in the "Infectious Diseases" category. However, we believe that this fact adds no systematic bias in the analysis of our data.

## Conclusion

In summary, we evaluated the worldwide trends of research productivity in the field of Infectious Diseases during an 8-year recent period. The results of this study showed a reassuring trend; the fact that developing world regions achieved a higher rate of increase of research productivity than the developed world regions. This is probably the result of increased awareness about the significance of infectious diseases in the developing world regions as well as improved infrastructure supporting research and development in these areas.

## Competing interests

The author(s) declare that they have no competing interests.

## Authors' contributions

MEF conceived the idea for the study; IAB and PIV collected the data; KP and AIK did the statistical analysis; IAB drafted the manuscript; all authors contributed in the writing and preparation of the manuscript. All authors read and approved the final manuscript.

## Pre-publication history

The pre-publication history for this paper can be accessed here:


